# Improved U-net network asphalt pavement crack detection method

**DOI:** 10.1371/journal.pone.0300679

**Published:** 2024-05-31

**Authors:** Qiong Zhang, Shanshan Chen, Yue Wu, Zhonghang Ji, Fei Yan, Shiling Huang, Yunqing Liu

**Affiliations:** 1 Changchun University of Science and Technology, Changchun, Jilin, China; 2 Jilin Provincial Science and Technology Innovation Center of Intelligent Perception and Information Processing, Changchun, Jilin, China; Shandong University of Technology, CHINA

## Abstract

Road crack detection is one of the important parts of road safety detection. Aiming at the problems such as weak segmentation effect of basic U-Net on pavement crack, insufficient precision of crack contour segmentation, difficult to identify narrow crack and low segmentation accuracy, this paper proposes an improved U-net network pavement crack segmentation method. VGG16 and Up_Conv (Upsampling Convolution) modules are introduced as backbone network and feature enhancement network respectively, and the more abstract features in the image are extracted by using the Block depth separable convolution blocks, and the multi-scale features are captured and enhanced by higher level semantic information to improve the recognition accuracy of narrow cracks in the road surface. The improved network embedded the Ca(Channel Attention) attention mechanism in U-net’s jump connection to enhance the crack portion to suppress background noise. At the same time, DG_Conv(Depthwise GSConv Convolution) module and UnetUp(Unet Upsampling) module are added in the decoding part to extract richer features through more convolutional layers in the network, so that the model pays more attention to the detailed part of the crack, so the segmentation accuracy can be improved. In order to verify the model’s ability to detect cracks in complex backgrounds, experiments were carried out on CFD and Deepcrack datasets. The experimental results show that compared with the traditional U-net network F1-score and mIoU have increased by 13.6% and 9.9% respectively. Superior to advanced models such as U-net, Segnet and Linknet in accuracy and generalization ability, the improved model provides a new method for asphalt pavement crack detection. The model is more conducive to practical application and ground deployment, and can be applied in road maintenance projects.

## 1. Introduction

In recent years, with the rapid development of the traffic industry and the expansion of the highway network, the importance of road maintenance has become increasingly significant. Many highways have problems such as cracks and potholes due to long-term heavy traffic load, complex external environment and degraded infrastructure performance. These damages shorten the life of the road and threaten the safety of motorists and pedestrians. Without timely repair of the cracked pavement, the problem may gradually worsen. Therefore, in order to ensure that the road is in good condition, timely detection of pavement cracks is a key part of road maintenance work.

With the development of defect detection technology, image processing technology is more and more applied to the identification and classification of defects, which has been studied in road crack detection, house crack detection and other fields. These topics have become the focus of modern road crack detection research, and some remarkable results have been achieved. Image recognition detection algorithms are mainly divided into traditional image segmentation methods and deep learning-based recognition methods. The traditional pavement crack recognition obtains the binarization image of the crack through the threshold segmentation method, extracts and analyzes the binarization features, and then describes the crack characteristics. The effect of this method depends on the image denoising process before pavement crack segmentation to a large extent. The commonly used denoising methods include spatial image denoising algorithm and frequency domain image denoising algorithm. Spatial denoising can reduce the effect of noise by processing image pixel values and using various filtering algorithms (such as median filtering and Gaussian filtering). Frequency domain denoising reduces noise by frequency domain transformation and filtering of the image. There may be isolated points or fractures in the image after noise removal. Relevant algorithms, such as minimum spanning tree or crack seed generation algorithm, can connect these discontinuous points. However, these methods are too complicated to handle some large data sets and are not suitable for this method.

For the crack image after noise removal, binary processing is usually carried out to obtain the crack features. This method is suitable for linear crack feature extraction with strong directivity (such as transverse crack or longitudinal crack), which can be extracted by edge detection algorithm. However, the road crack has no obvious directivity, so the edge detection algorithm is no longer suitable for crack detection. The traditional pavement crack recognition method has low generalization ability and needs to preprocess the image according to the crack morphology, and can not use the general algorithm to segment the crack morphology. The traditional road crack identification uses three steps of image denoising, image enhancement and image segmentation to extract pavement crack features, but can not achieve end-to-end crack detection.

With the development of deep learning technology, in recent years, researchers have used neural network models, which can directly rely on the input of the original image to complete end-to-end target recognition, without manual intervention in the segmentation process, so it is widely used in pavement crack detection. Compared with shallow models, deep learning is beneficial to feature extraction and recognition. However, classical deep learning models are primarily classification or recognition models for large and holistic datasets, such as AlexNet and GoogLeNet. In addition, it includes faster models such as the R-CNN. Due to the different shape and linear topology of cracks, if these deep learning models are directly applied to road crack detection, detection omissions and misjudgments will easily occur. In road crack detection, cracks are easily affected by complex road condition information such as grain size and marked edge, which brings difficulties to crack segmentation and effective identification. Still, there are some limitations to the current study, including the possibility that deep learning methods may be less sensitive to small cracks, and continuity challenges when dealing with complex irregular cracks.

To adapt to these challenges, this study aims to explore and develop new deep learning frameworks to improve the accuracy and efficiency of pavement crack detection. Our approach will focus on sensitivity to image detail and the ability to handle narrow cracks in the hope of providing a more reliable and practical solution for road maintenance. However, there are several limitations to the above research:

(1) Due to insensitivity to image details, previous deep learning methods are prone to produce false positives for fissures and expand the detection range of cracks’ edges.

(2) When dealing with irregular cracks, local branches and end discontinuities often appear, which is an obstacle to pixel segmentation.

In order to ensure accurate detection of asphalt pavement cracks, this paper proposes an improved U-net asphalt pavement crack detection method, aiming at the problems that the traditional segmentation network has less detailed information on crack profiles, difficulty in identifying narrow cracks and inaccurate segmentation edges in pavement segmentation, resulting in low recognition accuracy. Three contributions are proposed:

In this paper, VGG16 is used as the backbone network to extract features, and the obtained multi-scale features are passed through the Up_Conv module improved in the coding process, so that the network can capture and use higher level semantic information while maintaining a larger size feature map, and output more accurate crack contour details.

In this paper, Ca attention mechanism is introduced in skip connection to help the model pay more attention to the crack area, improve the accuracy of crack detection by enhancing crack information and suppressing background noise, and enhance the robustness of crack detection by adapting to cracks of different sizes and depths.

DG_Conv module and UnetUp module are introduced in the decoding process, which not only ensures the accuracy of the model, but also reduces the calculation amount and parameter number of the model, which helps to improve the convergence speed and performance in the training process. Compared with the traditional method, the algorithm can effectively prevent the problems of local branch and endpoint discontinuity.

In conclusion, the purpose of this research is to accurately detect cracks in the road under more realistic conditions, and to accurately detect the detailed parts of cracks. The framework is compared with several existing methods to verify its accuracy. The comparison results show that the improved U-net has higher crack segmentation accuracy and is more effective than the traditional algorithm in preventing local branches and end discontinuities. The research is organized as follows: Section 2 introduces the details of implementation; Experimental results and discussion are in section 3; Concluding observations are provided in section 4.

## 2. Related studies

Asphalt concrete pavement has been widely used in road construction in our country because of its high performance, and has become the main road paving material. However, with the continuous increase of traffic flow, the appearance of pavement cracks [1] has become a common phenomenon, which has a negative impact on the integrity and service life of the pavement. Traditional manual inspection methods are inefficient in terms of labor intensity, time consumption and cost. With the development of technology, image processing technology has become an effective means to detect pavement cracks.

In the past studies, Shi Y’s team [2] and Zou Q’s team [3] proposed a variety of image processing techniques based on feature extraction, and achieved certain results. These techniques usually rely on integration channel features, luminance elevation models and some classical image processing algorithms, such as region growth, edge detection and threshold-based methods [4–8]. Although these methods are more efficient and accurate than manual inspection, they are susceptible to interference from image background, noise, shadows, and uneven illumination [9],which affects the detection accuracy.

To improve the accuracy and dependability in identifying cracks, researchers have developed intricate manual features, including wavelet attributes [10], Local Binary Patterns (LBP) [11], and histograms of oriented gradients [12]. Following this, the adoption of more elementary machine learning techniques, such as Support Vector Machines (SVM) [13] and Random Forest (RF) [14], has been on the rise for crack detection tasks, delivering substantially superior results.

Despite the contributions of these methods to the field of crack detection, the feature extraction process is quite subjective. Moreover, these approaches struggle to differentiate between actual cracks and other irregularities like split joints and noise in complex roadway scenarios, leading to detections that are not always precise.

With the development of deep learning technology, in recent years, researchers have used neu ral network models, which can directly rely on the input of the original image to complete end-to-e nd target recognition without manual intervention in the segmentation process. Therefore, it has been widely used in pavement crack detection [15–18].The recognition of pavement cracks by deep learning method mainly goes through three stages: In the first stage, CNN sliding window technology is used to classify cracks [19]; In the second stage, the positioning frame technology was developed to realize the location of pavement cracks; In the third stage, pixel level semantic segmentation technology [20] was adopted to explore the extraction method of the precise shape of pavement cracks [21]. The advantage of semantic segmentation is that it can extract the target in the image pixel by pixel, complete the segmentation of the target shape, and then realize the target semantic analysis. The supervised learning is used to complete the concentration and reduction of the target information, and the continuous target shape is obtained. In view of the above advantages, semantic segmentation models including FCN [22]、U-net [23]、Segnet [24] and DeepLab [25]codec-decoding structures, have been continuously applied to the detection of pavement crack diseases.

The Res2Net proposed by Gao et al. [26] is an improvement on the basis of ResNet, replacing the original structure of a single convolutional kernel with a set of convolution and adding multiple residual structures within a single residual block. This residual-like structure can obtain feature combinations with different numbers and receptive fields, increase the receptive fields of each layer of the network, and obtain richer context information. However, this method ignores the applicability of the model, and in some complex scenarios, the details of road cracks are easy to be omitted, resulting in inaccurate detection results and affecting the performance of crack detection.

In the crack detection task, the image contains interference factors such as light, water stain and shadow, and background noise will cause interference to the detection, confusing the background and crack. Therefore, the existing technology introduces more attention mechanism to suppress noise, extract more crack information, and improve the crack detection performance.

Fu et al. [27] proposed a dual attention network (DANet) that adaptively integrates local features with their global dependencies, and its location attention module and channel attention module model the semantic interdependence of space and channel dimensions respectively. Although the introduction of attention mechanism can improve the applicability of the model to a large extent, However, the detection accuracy cannot be guaranteed, so researchers improve the performance of crack detection by improving the basic network.

In recent years, U-net model has been recognized as a leader in semantic segmentation tasks due to its multi-feature fusion design, which was initially used in biological cell segmentation and later widely used in medical image processing and other fields [28,29]. U-net has unique advantages for small sample image analysis and can achieve pixel-level segmentation of cracks with fewer training datasets. The improved models of U-net such as Crack U-net also show great potential in the high-precision identification of pavement cracks [30]. Still, there are some limitations to the current study, including the possibility that deep learning methods may be less sensitive to small cracks, and continuity challenges when dealing with complex irregular cracks. Wang et al. [31] proposed an I-UNet road crack network model, which uses cavity convolution to expand receptive field, and experiments show that the I-UNet network model is more robust than the U-Net network model. Shi [32] et al. used the VGG-Unet model to carry out defect detection on the images after compression and segmentation and clipping respectively, and the clipping and segmentation effect was better for the defects of large size, but not good for the defects of small size. Yang [33] et al. ’s encoder in the Unet model adopted ResNet residual network for feature extraction, and the detection effect of Unet model using residual network as encoder was improved. Models used for other tasks are not fully applicable to crack detection in complex environments. Considering the crack problem in the real pavement scene, the road is easy to be disturbed by the outside world and form irregular crack pavement in practical application. Aiming at irregular cracks and non-obvious cracks, this paper proposes an improved U-net network to improve the detection accuracy of road cracks by improving the detection ability of small cracks.

## 3. Related work

### 3.1 Improved overall structure of U-net network model

U-net network is mainly composed of encoding and decoding process, which effectively combines the down-sampling path to capture context information, and the up-sampling path to obtain accurate positioning information. Its unique "U" shape structure allows us to use feature maps at various resolutions, which is essential for obtaining richer spatial information as well as detecting multi-scale road cracks.

In this study, this paper proposes a road crack detection method based on deep learning, which takes the classic VGG16 network as the backbone of feature extraction, and uses its powerful feature coding capability to obtain a set of richly layered crack feature map. In particular, five different levels of crack feature maps are extracted from the VGG16 network, corresponding to different receptive fields and levels of detail, and these crack feature maps provide key visual clues for the crack detection task of the improved U-net network.

To solve the problem that important spatial information in cracks may be lost due to the common down-sampling operation in U-net architecture, we adopt the custom Up_Conv module. The Up_Conv module realizes down-sampling through maximum pooling and subsequent Block blocks. Compared with traditional pooling, the addition of Block blocks enables the model to learn more abstract and expressive feature representation while reducing dimension. In addition, we have incorporated the Ca attention mechanism into the model, which consists of two flows: Maximum pooling and average pooling capture the global maximum and average response, respectively, and re-inject this global information into the fracture feature map via 1x1 and 3x3 convolutional networks. In this way, the model can focus more precisely on the fracture region and effectively suppress interference in the background.

In the up-sampling phase, we introduce the DG_Conv module to replace the traditional direct convolution or up-sampling operations. GS convolution in DG_Conv is a grouping convolution structure, which strengthens the context correlation in the crack feature map and helps the model retain more semantic information when enlarging the crack feature map. This is particularly important for the task of preventing local branch and endpoint discontinuity in crack detection, because it involves accurate localization and segmentation of small and complex cracks.

**Fig 1:**
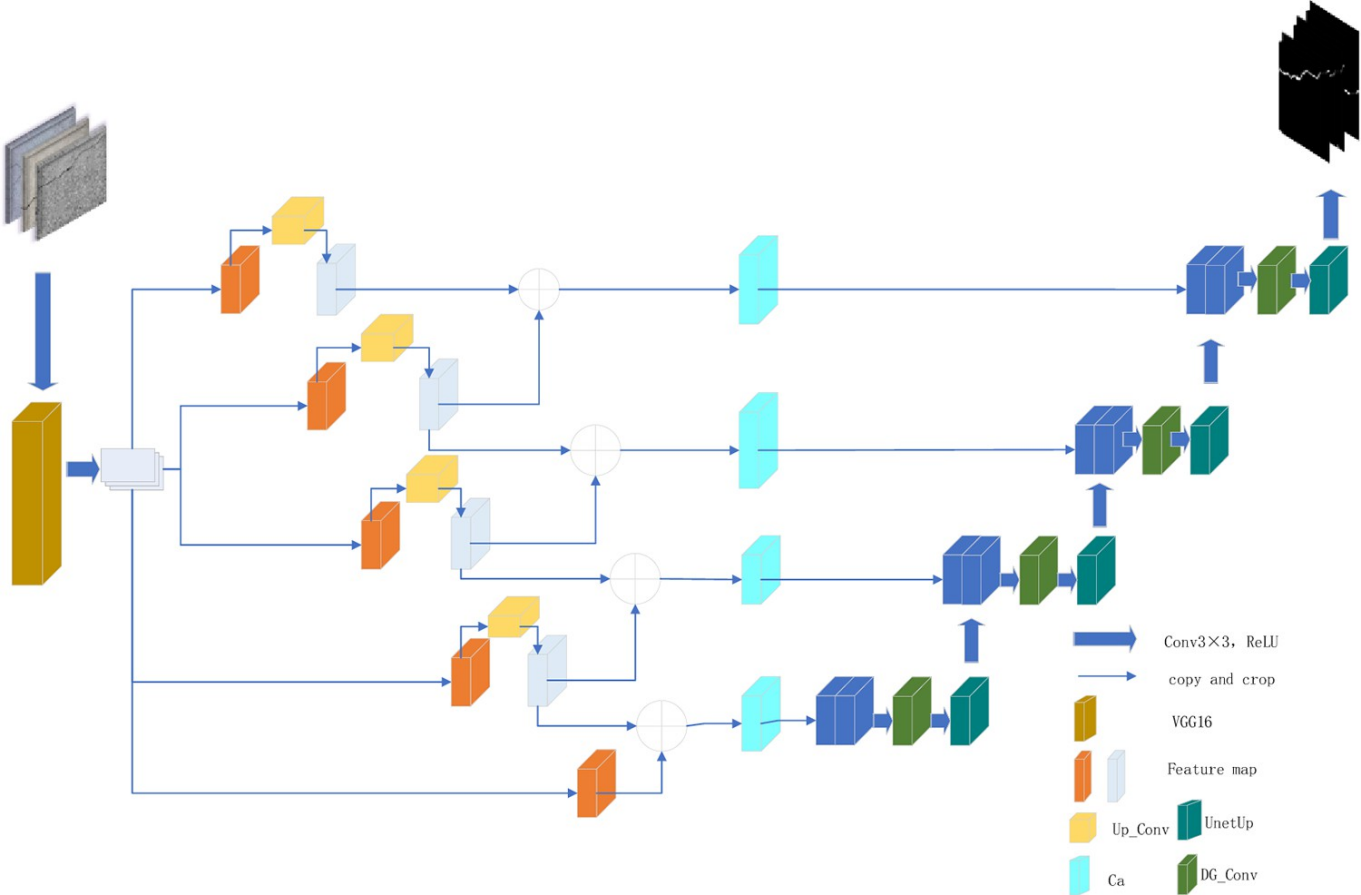
Improved U-net network structure diagram.

In addition, we designed the UnetUp module, which further extracts the up-sampled features through two consecutive 3x3 convolution layers to enhance the model’s learning of crack edges and textures. After each 3x3 convolution, GELU activation function is used, this unsaturated activation function can bring smoother gradient changes than ReLU, which helps to learn more delicate; Crack feature detail. At the sametime, the bilinear interpolation up-sampling method ensures the continuity and smoothness of the feature map in the amplification process, and avoids the artifacts that may be introduced by the common up sampling method. Finally, a 1x1 convolution layer is used to map the fused high-level feature maps into the predetermined class space, and the crack probability distribution map of each pixel is output. Through this series of well-designed modules, our approach significantly improves the accuracy of road crack detection in terms of crack continuity, detail retention, and robustness to complex backgrounds. These characteristics make the proposed model not only effective under laboratory conditions, but also have high application value in practical road maintenance engineering.The complete structure diagram is shown in Fig 1.

### 3.2 Improved U-net feature extraction network

In the task of road crack detection, the traditional U-net architecture has certain limitations in feature extraction capability due to its relatively shallow network level. This limitation usually leads to the loss of crack detail information, especially when dealing with cracks of different scales. To compensate for this, researchers often adopt strategies to increase the depth and width of the network. However, simply deepening and broadening the network structure can increase the characterization ability of the model, but it also leads to an increase in computational burden.

To solve the above problems, this paper proposes a strategy using VGG16 network as the backbone of feature extraction, which uses convolutional kernels with different receptive fields to capture crack features and improve the adaptability of the network to different scales of semantic information. By splicing multi-scale features on the basis of VGG16 network, the proposed method can not only retain rich fracture feature information, but also avoid the performance loss of traditional U-Net when processing cracks of different scales.

Further, to solve the problem that the multi-scale features output by VGG16 may still be limited in the expression of fracture details, a novel Up_Conv module is introduced in this study, which can effectively strengthen the features extracted from VGG16 by combining the maximum pooling and Block modules. The Up_Conv module is unique in that it transforms the traditional down-sampling process into a feature-enhanced up-sampling process. In this module, the maximum pooling part is responsible for extracting local features of this response, while the Block module makes use of depth-separable convolution blocks for feature abstraction and refinement. This design not only enhances the network’s ability to learn the crack features, but also reduces the channel dimension through 1x1 convolution, reducing the number of parameters and computational complexity of the model. Finally, the output of maximum pooling and Block module are added together, and the final feature map is obtained by subsequent 1x1 convolution and activation function.

**Fig 2:**
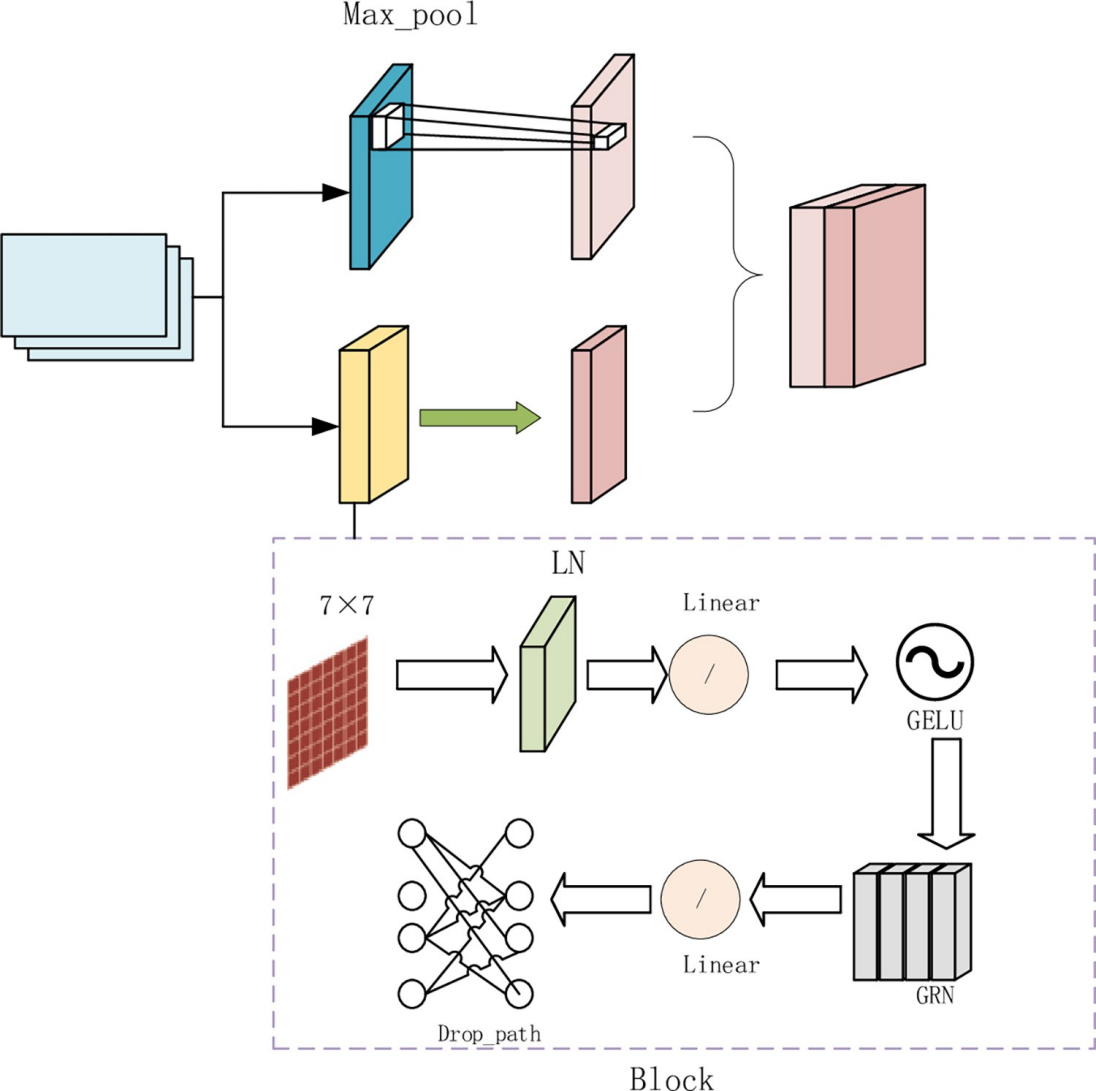
Up Conv module.

The Block module adopts the design of depth-separable convolution block, which combines depth-separable convolution, layer normalization, point convolution, activation function, global response normalization, second point convolution, and Drop_Path operation. This design aims to reduce the computational complexity while extracting more abstract features, so as to capture and utilize higher-level semantic information while maintaining a relatively large feature map size, especially for the retention of spatial information that is crucial in crack detection tasks. The improved Up_Conv module construction is shown in Fig 2.

Another big advantage of the Block module is its deep separable convolution structure, which breaks down traditional convolution operations into two sub-steps of layer by layer processing and point by point processing. This decomposition allows the module to process larger feature graphs with fewer parameters, thus extracting more spatial and channel dimension information without significantly increasing the computational burden. Combined with layer normalization, activation function, global response normalization, and Drop_Path operations, the Block module further improves the characterization power of features and the generalization ability of models.

### 3.3 Feature fusion network based on Ca attention mechanism

In the deep learning application of road crack detection, although the traditional U-net codec structure can effectively achieve multi-scale feature extraction, there are some limitations. Specifically, the shallow part of the network can generate feature maps with high resolution, which is conducive to the retention of detailed information; While the deep part of the network can extract rich semantic information, but its resolution is lower. In this framework, the function of skip connection is to simply splice the feature map of the encoder part with the corresponding feature map of the decoder part to recover some spatial information. However, this method does not consider the importance difference between the features, which may lead to the limitation of key crack features in the transmission process, and thus the loss of detailed information in the final crack detection result. Especially in changing environments and different scenes, the detection performance may be significantly affected.

In order to solve this problem and strike a balance between retaining high-resolution detail information and rich semantic information, this study proposes an attention-mechanism-based feature fusion strategy, especially in the skip connection part. In the feature fusion between encoder and decoder, we introduce the Ca attention mechanism, which includes two important components: channel attention mechanism and spatial attention mechanism. The channel attention mechanism is designed to capture the importance of different channels in the feature map, and then pay attention to the scale information of crack features. The spatial attention mechanism focuses on the spatial information within each channel, which enhances the dimensional attention to the fracture features. By combining these two attention mechanisms, our model is able to effectively distinguish and highlight fracture areas while suppressing background noise, and thus more accurately reflect the fracture details in the feature map.

#### 3.3.1 Channel attention mechanism

The channel attention mechanism extracts the spatial information of crack features by Max_Pool and Avg_Pool to generate two spatial context features and then input the two features *F*_*avg*,*C*_ and *F*_*max*,*C*_ into a Multi-Layer Perceptron with hidden layers., is calculated and added into the network, and the channel attention is obtained by Sigmoid function calculation *M*_*C*_. In order to reduce the number of parameters, the hidden layer size of MLP is set to *C*_*r*_ × 1 × 1, where r is the compression ratio. Channel attention is calculated as follows:

$$\begin{array}{l} M_{C}(F)=\sigma (MLP(A\mathrm{vg}\_\mathrm{Pool}(F))+MLP(Max\_Pool(F)))\\ \;=\sigma (W_{1}(W_{0}F_{avg,C})+W_{1}(W_{0}F_{\max,C})) \end{array}$$ (1)

Where:*σ* represents the Sigmoid function: $W_{0}=\frac{C}{r}\times C;W_{1}=C\times \frac{C}{r}$。

This attention map selectively amplifies informative channels while attenuating the less relevant ones by applying learned weights to each channel. Implementing this mechanism allows the network to dynamically fine-tune feature representation, thereby enhancing performance across tasks such as image classification and, specifically, the extraction of spatial information pertinent to crack detection. The refined focus on discriminative features not only ensures greater task accuracy but also maintains model efficiency by optimizing the number of parameters involved.

#### 3.3.2 Spatial attention mechanism

Spatial attention pays attention to the location information of the same channel in the fracture characteristics, and complements the discontinuous information of the fracture end points of the channel attention. The features of the input spatial attention(*F*’ = *C*×*H*×*W*) are summed by average pooling (*F*’_*avg*_ = 1×*H*×*W*)and max pooling(*F*’_max_ = 1×*H*×*W*), and then they are concatenated based on channels(*F*’_*avg*+max_ = 2×*H*×*W*). The resulting feature map is convolved using 7×7 convolution kernel with channel number 1, and compressed into a feature map with channel number 1. The features of spatial attention *M*_*s*_ = 1×*H*×*W* are obtained by Sigmoid function operation. The calculation method of spatial attention force is as follows:

$$\begin{array}{l} M_{S}(F^{'})=\sigma \{f^{7\times 7\{[AvgPool(F^{'})+MaxPool(F^{'})]\}}\}\\ \;=\sigma \{f^{7\times 7}[{F^{'}}_{avg}+{F^{'}}_{\max}]\} \end{array}$$ (2)

Where: σ represents the Sigmoid function; *f*^7×7^ Represents a convolution operation with a

filter size of 7×7.

#### 3.3.3 Fusing channel attention and spatial attention mechanism

**Fig 3:**
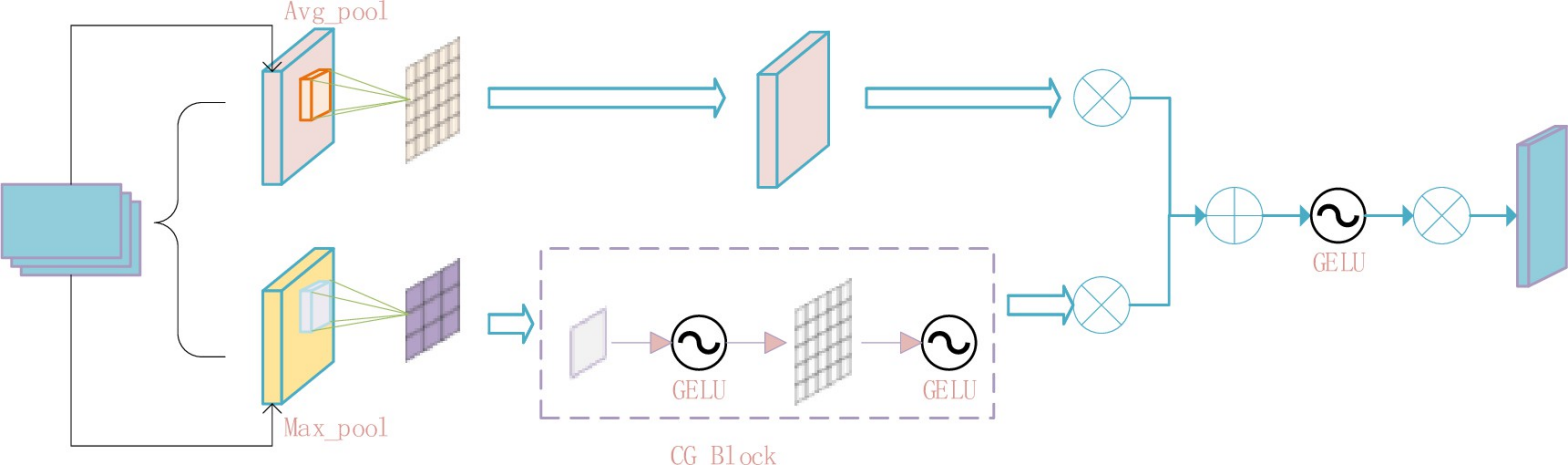
Ca attention mechanism.

The Ca attention module proposed in this paper includes two parts: average pooling and maximum pooling. After average pooling, 5×5convolution is carried out, and then multiplication is performed with input features. After maximum pooling, two convolution operations are carried out, and then multiplication is carried out with input features, the results obtained from the two pooling are added and fused, and the final output feature map is obtained by multiplication and fusion with input features after sigmoid activation function. Fig 3 shows the structure diagram of Ca attention mechanism.

In the process of implementing the above attention mechanism, this paper adopts global average pooling and maximum pooling operations for the channel attention module to generate a description of the statistical characteristics of the channel, and then uses the full connection layer to learn the dependencies between the channels, so that the network can adaptively enhance the response to important channels. For the spatial attention module, this paper generates spatial attention graphs by performing convolution operations on the feature graphs to highlight the more spatially critical regional features. By combining channel attention with spatial attention, we realize dynamic weight adjustment in the process of feature fusion to retain more continuous information of crack end points enhance the generalization ability of the model to complex road environments. This innovative combination of Ca attention modules not only optimizes the effectiveness of feature fusion, but also improves the overall performance of road crack detection.

To sum up, the feature fusion method based on Ca attention mechanism proposed in this paper significantly enhances the model’s ability to capture key crack information, effectively improves the detection effect in different environments and scenarios, and achieves an effective balance between details and semantic information for road crack detection under the framework of deep learning. This strategy provides a reliable and efficient solution for automatic crack detection under complex road conditions.

### 3.4 DG_Conv network and UnetUp network in improved U-net network

In the U-net structure, the up-sampling part is very important to recover the high-resolution detail information of the image. However, the traditional U-net faces challenges in the up-sampling process: since the skip connection only transmits the original features in the encoder, there is no effective refinement and optimization of the features, so the high-resolution feature map reconstructed at the decoder end often lacks fine detail information and clear edge definition. Especially in tasks that require precise details such as road crack detection, this loss of information can cause the accuracy of crack identification to be seriously affected.

To solve this problem, two modular solutions are proposed in this study: DG_Conv module and UnetUp module, which aim to enhance the feature recovery and refinement capability of the sampled part on U-Net, so as to improve the crack detection performance.

**Fig 4:**
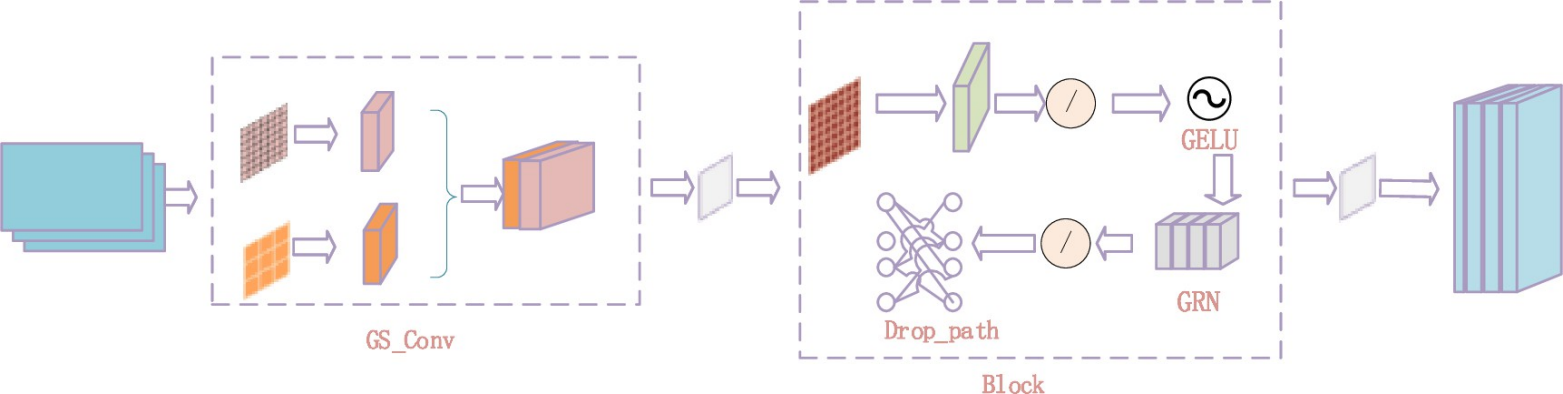
DG_Conv module.

The DG_Conv module is elaborately designed and consists of group convolution (GS_Conv), two 1×1 convolution, and a Block module, all of which work together to effectively improve the efficiency of feature extraction and fusion. As shown in Fig 4, the GS_Conv module first uses traditional convolution to capture the initial feature representation, and then uses deep separable convolution to further refine the features and reduce the number of parameters. Finally, the results of these two convolution operations are optimized and combined by channel rearrangement technology, so that the features extracted from different convolution kernels can complement each other, so that the semantic information of cracks and its surrounding can be preserved more comprehensively without increasing the computational burden.

**Fig 5:**
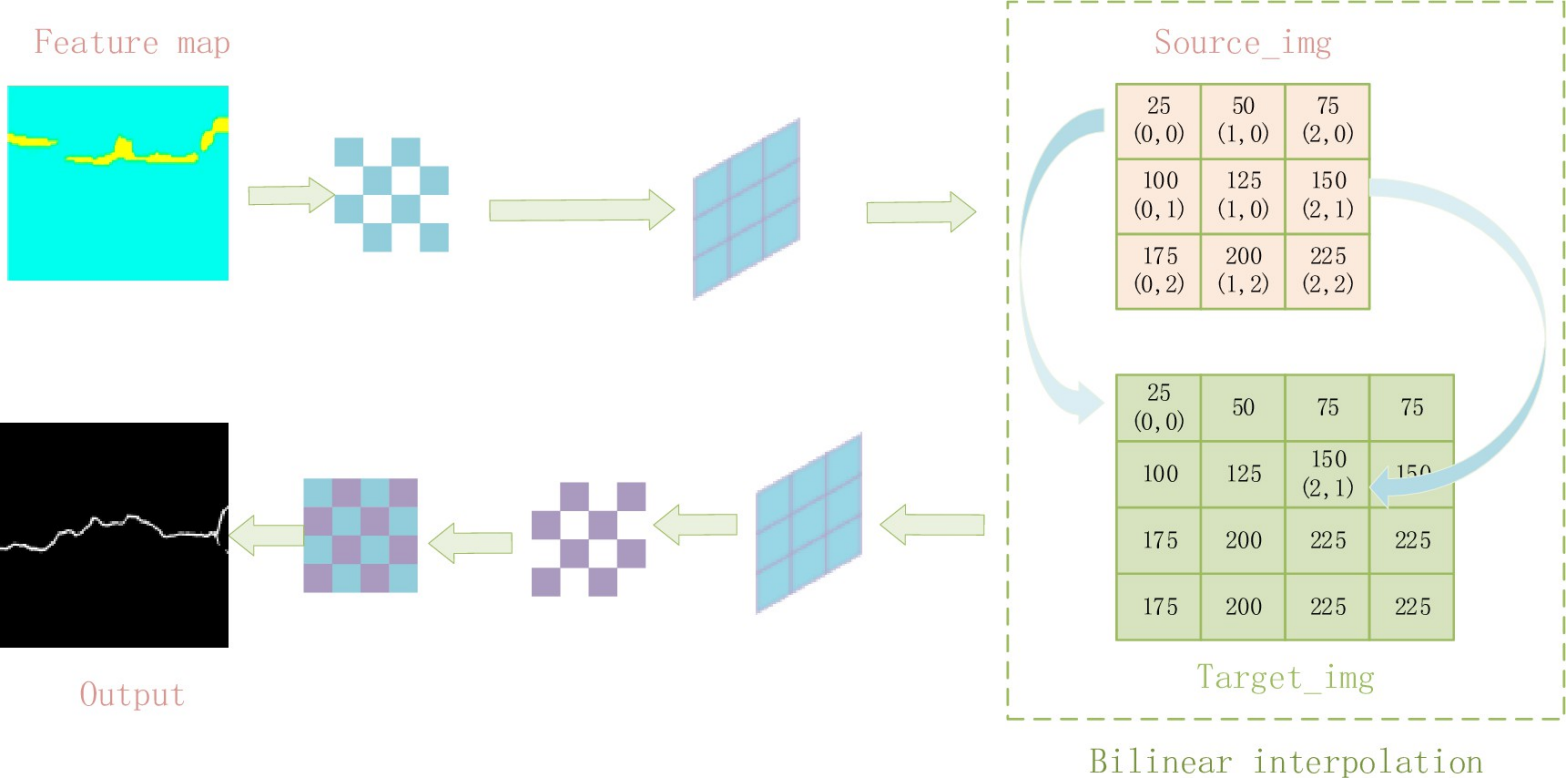
UnetUp module.

The UnetUp module focuses on the feature quality after up-sampling, and combines up-sampling and convolution operations to fine recover the feature map. As shown in Fig 5, by using two 3x3 convolution layers immediately after bilinear interpolation up-sampling, the UnetUp module can effectively compensate for the loss of detail information in the up-sampling process and enhance the model’s ability to recognize the crack edge. This modular design not only improves the accuracy of crack detection, but also enables the model to better deal with local discontinuities and small cracks.

By integrating the DG_Conv module and the UnetUp module into the up-sampling phase of U- Net, we have significantly improved the traditional up-sampling mechanism. This improvement not only achieves significant performance gains in fracture feature recovery, but also in the sharpness of fracture edges and overall segmentation accuracy. The experimental results show that compared with the traditional U-net, the improved model has superior crack detection performance under various complex road conditions, which fully validates the effectiveness and practicability of the proposed method. This research will have a positive impact on the development of automated road maintenance and crack detection technology in the future intelligent transportation system.

## 4.Experiment

### 4.1 Experimental environment configuration

The programming environment for the experiment is Python3.8 and torch version 1.10.8. The hardware environment is Intel I7-7800X CPU with NVIDIA GeForce RTX3090 graphics card with 24GB video memory and 16GB RAM. In order to prevent the model from falling into the local optimum during the training process, Adam optimizer was used to optimize the training.

### 4.2 Dataset

In order to verify the validity of the network model proposed in this paper, we introduce pavement crack data sets: CFD dataset and Deepcrack dataset.

1. CFD dataset

The dataset consists of 118 labeled crack images with a resolution of 480×320. In order to avoid overfitting and improve the generalization ability of the network, 590 crack images and their corresponding labeled images were generated on the basis of the original crack images by blurring, enhancing, decreasing, rotating 180 degrees and horizontal mirroring. After the expansion, the final dataset sample is 708, and 420 images are randomly selected to build a training dataset, 144 images to build a validation dataset, and 144 images to build a test dataset.

2. Deepcrack dataset

The dataset consists of 537 manually labeled pavement crack images, 300 of which were used for training and 237 for testing. In order to better train the model in this paper, the size of the training dataset is a crucial influence point. However, due to the relatively small scale of the current publicly available crack detection dataset, the accuracy of the model training will be reduced to a certain extent. Therefore, the dataset is expanded by means of data enhancement before training. Each image in the current Deepcrack training dataset is rotated 8 times by Angle and horizontal rotation, and the rotation angles are [45°、 90°、 135° 、180°、225°、270°、315° and 360°]. Finally, each image is expanded to 16 pieces, that is, the dataset is expanded to 16 times. At the same time, in order to enable the model to train and converge quickly, the image of the training dataset is scaled to 256×256.

### 4.3 Evaluation index

When evaluating the performance of deep learning models on semantic segmentation tasks, it is essential to adopt standardized and universally accepted evaluation metrics to ensure the objectivity and accuracy of the evaluation process. Therefore, this study will incorporate several key performance metrics commonly used in semantic segmentation research, including F1-score Recall ©, average crossover ratio (mean Intersection over Union, mIoU) and Precision (P). The formula is as follows:

$$\mathrm{Precision}=\frac{TP}{TP+FP}$$ (3)

Recall=TPTP+FN (4)

$$F1=\frac{2\times precision\times recall}{precision+recall}$$ (5)

$$mIoU=\frac{1}{k+1}\sum_{i=0}^{k}\frac{TP}{TP+FP+FN}$$ (6)

Since F1-score combines accuracy rate and recall rate, it can balance the relationship between them well, so this paper uses F1-score as the final result. The precision rate reflects the percentage of all samples predicted to be positive that actually turned out to be positive, while the recall rate measures the percentage of all positive that were correctly predicted to be positive. Ideally, we want the model to have both a high accuracy rate and a high recall rate, but in practice, raising one metric tends to lower the other. In order to fully measure the performance of the model, it is often necessary to find a balance between these two metrics. By evaluating accuracy and recall rates together, we can gain a deeper understanding of the underlying causes of a model’s declining performance and adjust strategies accordingly to meet specific challenges. The F1-score is the harmonic average of the accuracy rate and the recall rate, which is balanced when both are high, and is a valid indicator of the model’s performance as a whole.

On this basis, we also define four types of key quantities in the detection results: True Positive (TP), that is, the number of cracked pixels correctly identified as cracks; False Positive (FP), which is the number of background pixels wrongly identified as cracks; True Negative (TN), which is the number of background pixels correctly identified as background; And False Negative (FN), which is the number of cracked pixels wrongly identified as background. These quantities form the basis for evaluating the predictive performance of the model, allowing us to quantitatively analyze the accuracy of the model’s predictions.

### 4.4 Improve model performance evaluation

In order to verify the effectiveness of the proposed network, the proposed network, U-net, Segnet and Linknet were fixed in the same hardware environment and parameter initialization conditions, and tested on the CFD dataset and Deepcrack dataset respectively. The detection results of different networks on CFD dataset and Deepcrack dataset are shown in Figs 7 and 8. The red and yellow boxes in the figure are the differences between the detection of the basic network and other networks. It can be seen that for small cracks, the cracks detected by the original U-net network have a large gap with the real road surface, and even misdetection results may occur. If some shadow parts or differences in light and shade are identified as cracks, the detection accuracy and various indicators will decrease. In the figure, it can also be seen that compared with the original U-net, Segnet and Linknet can detect the correct cracks more obviously, but for some details like red and yellow frames, they are still different from the real road surface to some extent. However, the model proposed in this paper can not only correctly detect the real cracks, but also detect the real cracks. It can also detect narrow cracks, which is similar to the real road surface.

In addition, in order to objectively verify the advantages of the improved model, different algorithms were evaluated on two datasets using the evaluation indexes proposed in this paper. The results of the comparative experiment are shown in Table 1.

**Fig 6:**
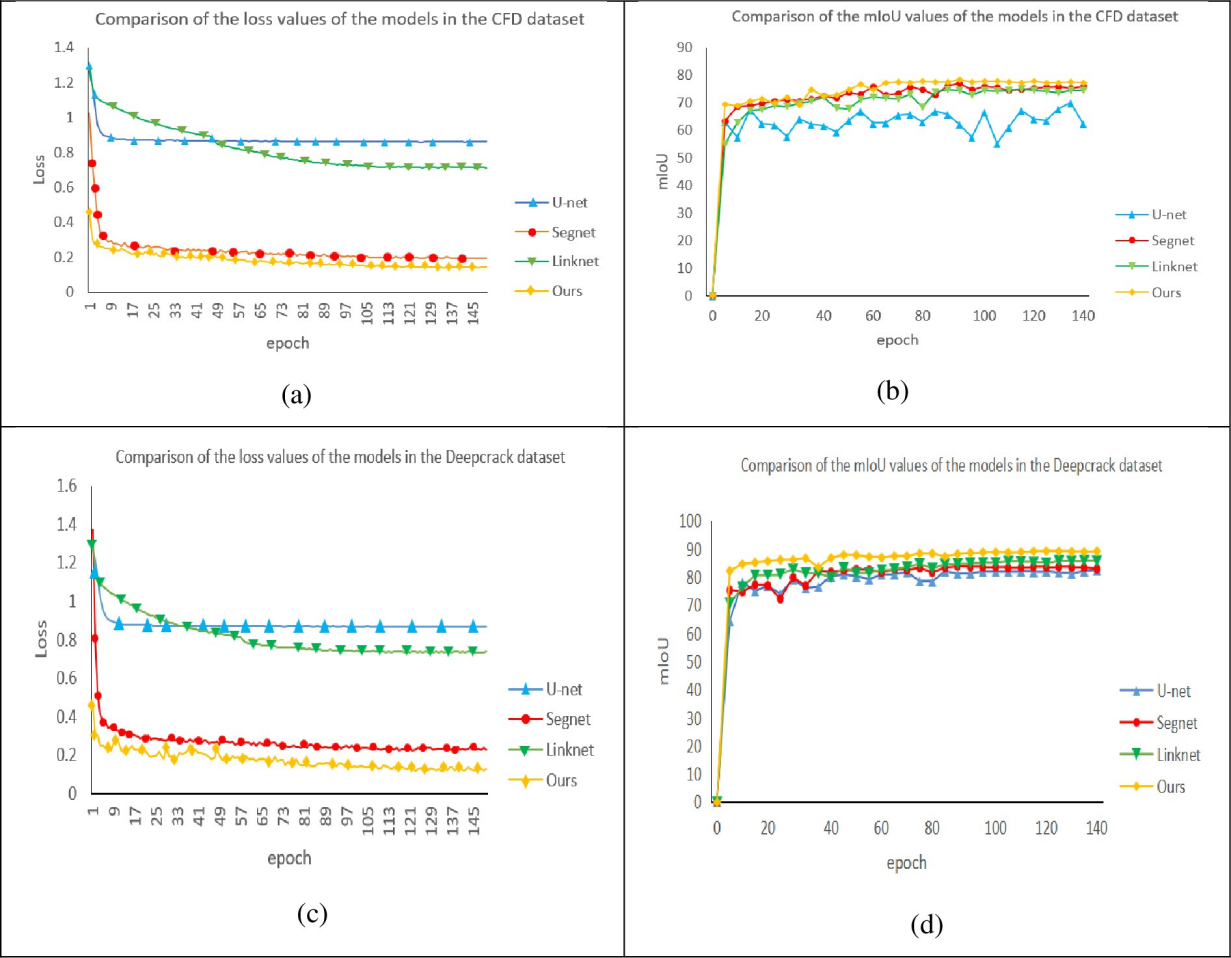
Comparison of model parameters of two datasets.

(1) Experimental results on CFD datasets: As can be seen from Table 1 and Fig 6(A) and 6(B), the proposed algorithm outperforms other algorithms under the same experimental conditions, with P and F1 reaching the highest and Loss reaching the lowest. Compared with U-net, Segnet and Linknet, it increased by 11.6%、8.7%; 3.5%、3.8%;5.5%、2.6%, the Linknet network reached its highest in R. In Fig 6(A), it can be seen that the loss value of U-net in the CFD dataset tends to be stable to about 0.88 after the tenth iteration, the Linknet network is second only to the U-net network to about 0.75, and the Segnet network can reach about 0.23, while the models proposed in this paper are lower than the above models, with the lowest reaching 0.162. In Fig 6(B), we can see the comparison of the mIoU values of the four networks in the CFD dataset, with the highest value of the U-net network reaching 68.5, the highest reaching of the Linknet network reaching 71.2, and the highest reaching the Segnet network reaching 72.5, while the model proposed in this paper is higher than the above four networks, reaching the highest of 78.4.

(2) Experimental results on the Deepcrack dataset: From Table 1 and Fig 6(C) and 6(D), it can be seen that under the same experimental conditions, the proposed algorithm is superior to other algorithms, with P, R, and F1 reaching the highest, and the Loss reaching the lowest. Compared with U-net, it increased by 1.8%、5.3% and 3.6%, compared with Segnet by 1.7%, 1.9% and 1.9%, and increased by 2.0%, 1.5% and 1.6% compared with Linknet. In Fig 6(C), it can be seen that the loss value of U-net in the Deepcrack dataset tends to be stable to about 0.87 after the tenth iteration, the Linknet network is second only to the U-net network to about 0.77, with the lowest reaching 0.138. In Fig 6(D), we can see the comparison of mIoU values of the four networks in the Deepcrack dataset, with the highest value of 80.3 for the U-net network, 82.5 for the Linknet network, and 81.2 for the Segnet network, while the model proposed in this paper is higher than the above four networks, reaching the highest of 89.5.

**Table 1:** Compare the results of experimental evaluation.

Network	CFD dataset			Deepcrack dataset		
	P	R	F1	P	R	F1
U-net	0.748	0.804	0.775	0.856	0.834	0.845
Segnet	0.829	0.838	0.824	0.857	0.868	0.862
Linknet	0.809	**0.865**	0.836	0.859	0.872	0.865
Ours	**0.864**	0.861	**0.862**	**0.874**	**0.887**	**0.881**

**Fig 7:**
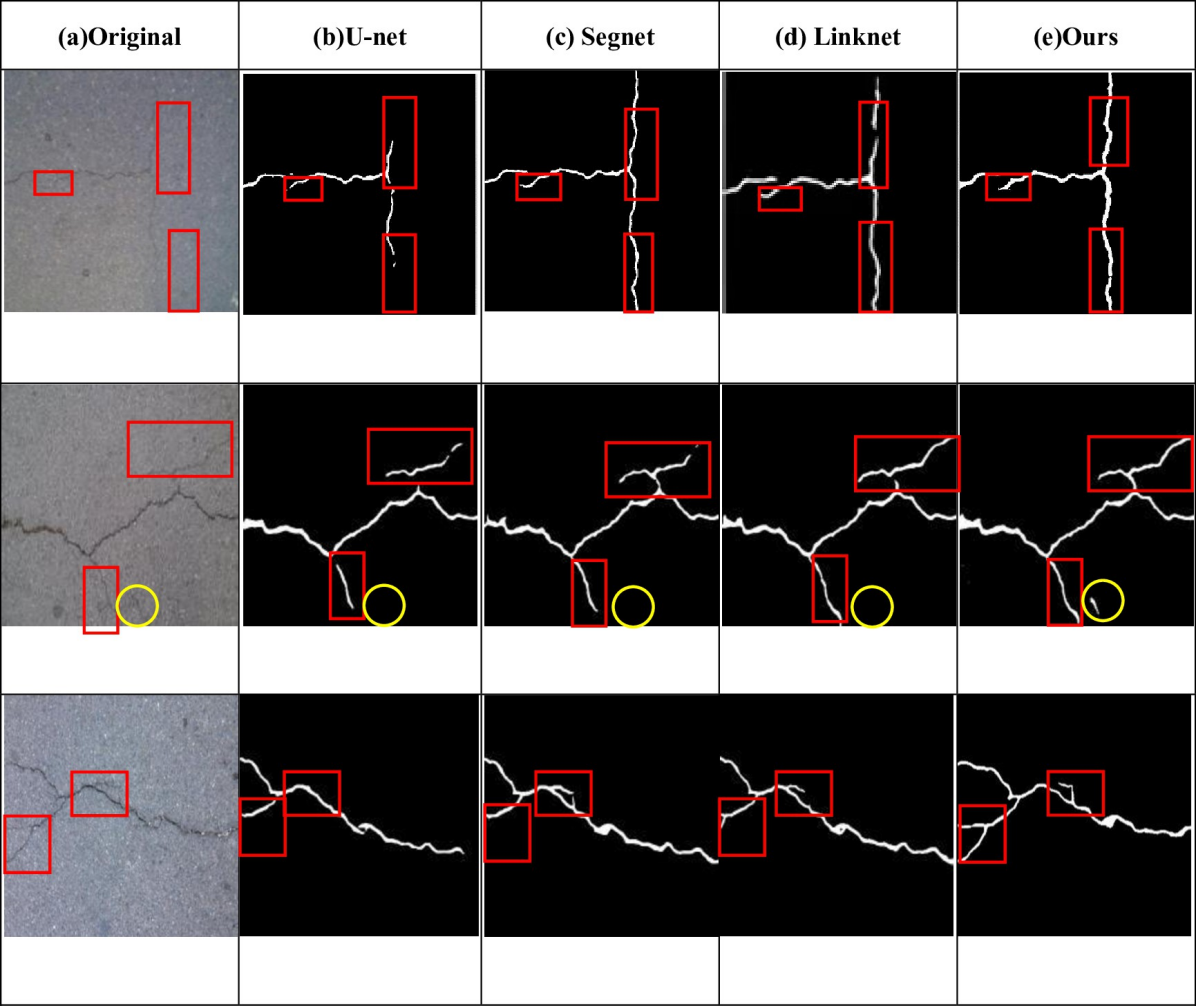
Images of detection results of different networks on CFD dataset.

**Fig 8:**
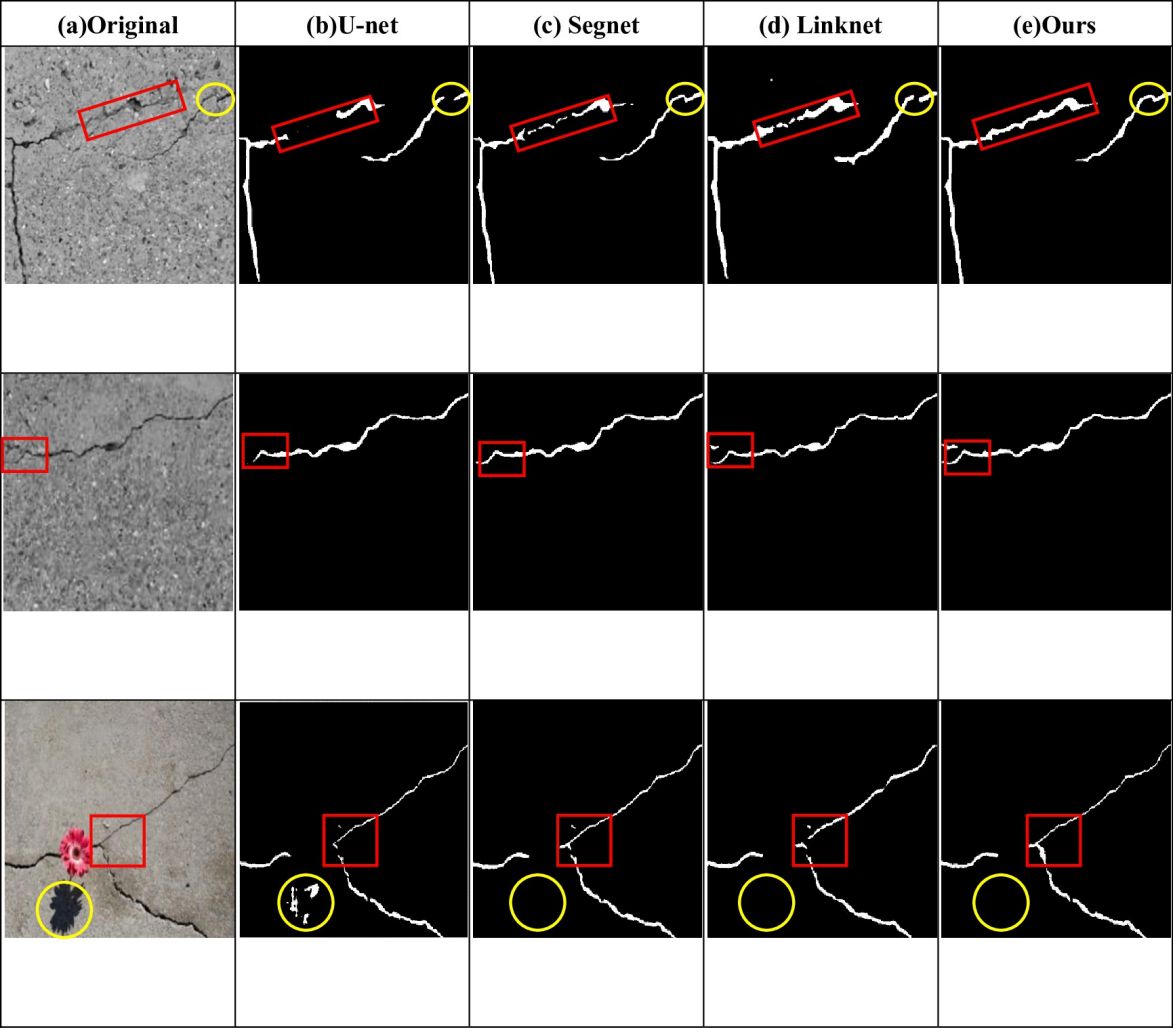
Image of detection results of different networks on Deepcrack dataset.

From the subjective point of view, the improved model proposed in this paper can better display the details of crack detection, and the prediction results are less affected by noise, which can reflect the real road conditions, as shown in Figs 7 and 8. Objectively, after testing two public data sets and comparing the detection conditions of different algorithms, it can be clearly concluded that the improved algorithm proposed in this paper is superior to several other algorithms. Table 1 and Fig 6 show that all indicators of the proposed algorithm are superior to other algorithms. Therefore, no matter from the subjective or objective point of view, the algorithm proposed in this paper can accurately extract the crack characteristics under complex background, and has a good detection effect.

### 4.5 Ablation experiment

In order to verify the influence of different network modules on crack detection performance, we separately added the Up_Conv module, Ca attentional mechanism module and DG_Conv module to the benchmark U-net for training, and conducted ablation experiments on the two datasets.

**Fig 9:**
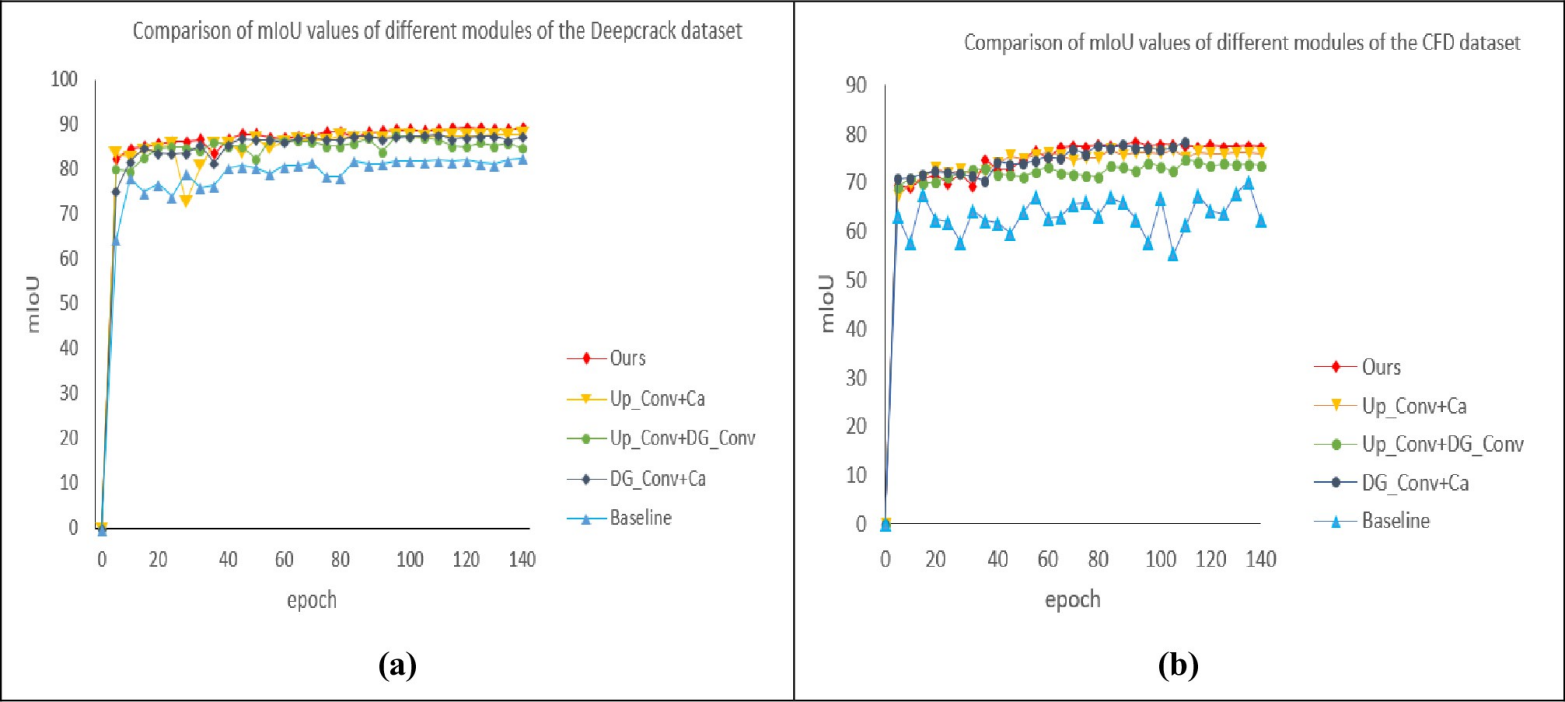
Comparison of mIoU values of different modules in the two datasets.

**Fig 10:**
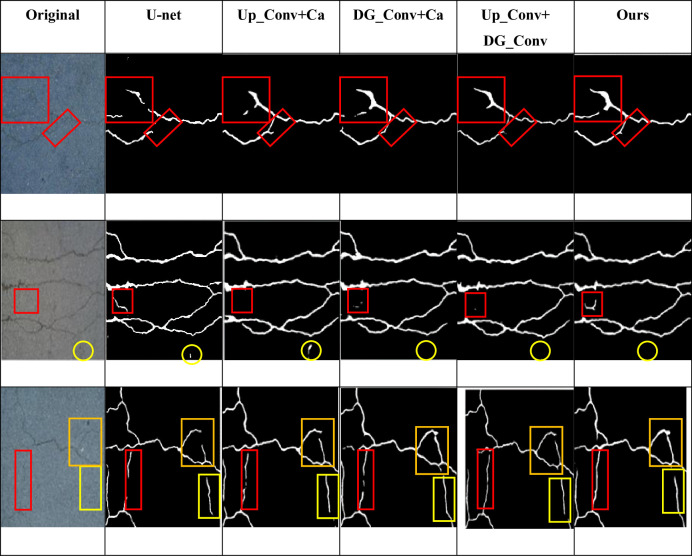
Compasion diagram of different modules in CFD datasets.

In the CFD dataset: As can be seen from Table 2, the segmentation module contributes to the performance improvement of the network. Among them, the DG_Conv module retains more semantic information in the crack through the GS convolution, which is helpful to locate the crack location information, and without adding the module (+Up_Conv+Ca), compared with U-net, it is 16.7%, 10.1% and 13.3% higher than that of U-net in P, R and F1, respectively, and P reaches the highest, and mIoU is increased by 8.3%, Up_Conv module obtains a larger receptive field for multi-scale feature processing when extracting features. Compared with U-net, the without Up_Conv module (+DG_Conv+Ca) was increased by 13.9%, 10.7% and 12.3% in P, R and F1, respectively, and the R was higher than that of the other four groups. Without Ca channel attention (+Up_Conv+DG_Conv), R is lower than the other three groups, and mIoU is lower than the above two groups. F1, Loss, and mIoU are all better than the top four groups in the basic network, and F1 and mIoU are 13.6% and 9.9% higher than those of U-net. The CFD dataset mIoU value pair is shown in Fig 9(A). It can be seen that with the increase of the number of iterations, the mIoU of the proposed model is higher than that of the above models, which verifies the effectiveness of the model. As shown in Fig 10, you can see the influence of different modules on the detection effect, in the second row, it can be seen from the original picture that the yellow circle part is not crack information, but the above model detects crack information, resulting in low accuracy, and the model proposed in this paper can correctly detect the real situation of the road, indicating that the model proposed in this paper is better than the other four.

**Table 2:** Ablation experiments of CFD dataset.

Network	P	R	F1	mIoU	Loss
Baseline	0.713	0.754	0.733	0.685	0.861
+Up_Conv+Ca	**0.880**	0.855	0.866	0.768	0.226
+DG_Conv+Ca	0.852	**0.861**	0.856	0.749	0.297
+Up_Conv+DG_Conv	0.826	0.852	0.840	0.725	0.353
Ours	0.879	0.860	**0.869**	**0.784**	**0.162**

In the Deepcrack dataset: As can be seen from Table 3, the segmentation module contributes to the performance improvement of the network, and R and P are increased by 5.8% and 5% compared with U-net, F1 is increased by 5.4%, and mIoU is increased by 7.4% compared with U-net without DG_Conv (+Up_Conv+Ca), which is second only to the indicators of this model. No Up_Conv (+DG_Conv+Ca) P, R, and F1 values were lower than those of the previous model, which were 4.1%, 4.9%, and 4.6%, respectively, compared with U-net, and were also lower than those of this model. No Ca (+Up_Conv+DG_Conv) was lower than the three groups of ablation results, but the indicators were better than the original U-net network model.

**Fig 11:**
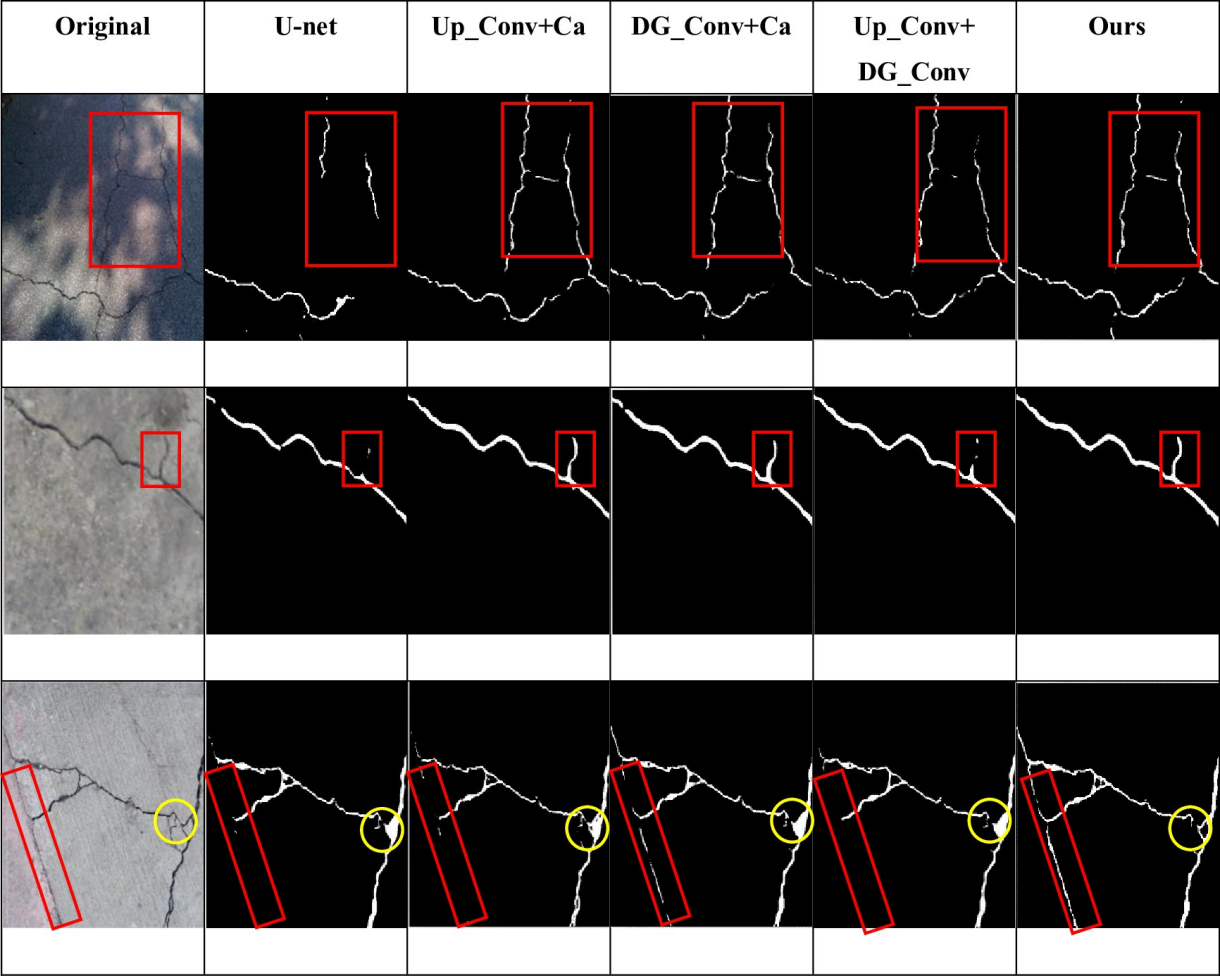
Comparison of different modules in Deepcrack dataset.

In this paper, the F1 of the model reaches the highest, with an increase of 5.9%, and the mIoU also reaches the highest, with an increase of 9.2%, and the loss is the lowest compared with the previous four groups, indicating that the performance of the proposed model is better than that of the original model. Fig 9(B) also shows that the mIoU value of the model is higher than that of the other four groups. Fig 11 shows the detection effect, and the red and yellow boxes can show the difference between the model in this paper and the others.

**Table 3:** Ablation experiments of Deepcrack dataset.

Network	P	R	F1	mIoU	Loss
Baseline	0.856	0.834	0.845	0.827	0.867
+Up_Conv+Ca	0.871	0.882	0.876	0.877	0.161
+DG_Conv+Ca	0.862	0.873	0.868	0.873	0.172
+Up_Conv+DG_Conv	0.864	0.867	0.865	0.868	0.186
Ours	**0.874**	**0.887**	**0.881**	**0.895**	**0.138**

## 5. Conclusion

In this paper, an improved U-net network based on U-net network is designed for the crack detection of asphalt pavement with complex background. Aiming at the problems such as large number of parameters of deep learning model and difficulty in capturing feature details, an improved U-net model is proposed for the identification of road cracks. In the process of U-net model coding, Up_Conv module is used to replace the up-sampling process, which enhances the multi-scale features and improves the multi-scale information learning ability of the model. In the decoding process, DG_Conv and UnetUp modules are used to improve the learning ability of the model to crack information and the resistance ability to non—crack information. The model can quickly realize the crack detection in the UAV image, and presents a new choice for the crack detection task of asphalt pavement. Through the test on the crack opensource dataset, it is concluded that the model performs better than the common deep learning model and the basic U-net in crack detection under various scenarios. The main advantages areas follows:

1. Feature extraction through the VGG16 network can aggregate more scale information, and better adapt to crack detection in scenes with less obvious features, different lighting and complex background.
2. By distinguishing cracks and background noise through the designed attention mechanism, the detailed part of cracks can be more prominent and better reflect the real situation of the road surface.
3. The improved coding process is essentially an up-sampling process, which cannot only extract more abstract features in the image while reducing the computational complexity, but also enable the network to capture and utilize higher-level semantic information to a large extent, especially in crack detection, paying more attention to spatial information.
4. In the process of the improved decoding, the module not only retains more semantic information but also has a lower number of model parameters, which reduces the complexity and ensures the accuracy.

However, although this improved U-net network can handle some small cracks and improve the crack detection accuracy, there are still some limitations. Because the real pavement is affected by the environment, there will be a large number of cracks with different lighting, occlusions and damage, which have a crucial impact on the crack detection accuracy. Most of the crack features in the model training process are free from the above interference, and it is difficult to ensure that the model is suitable for the above crack features. Therefore, expanding the data set is necessary to improve the accuracy of crack detection. The crack image with interference is used as the training model to ensure the generalization ability of the model. The original crack data can also be preprocessed to obtain images that meet the experimental requirements. In addition, due to the lack of camera calibration, it is difficult to obtain the physical parameters of the crack, such as length, width and area. However, accurate image crack detection can further multi-dimensional data fusion of images with other high-precision information, such as radar, laser, and GPS data, to compensate for the shortcomings of different information models.
